# Lundep, a Sand Fly Salivary Endonuclease Increases *Leishmania* Parasite Survival in Neutrophils and Inhibits XIIa Contact Activation in Human Plasma

**DOI:** 10.1371/journal.ppat.1003923

**Published:** 2014-02-06

**Authors:** Andrezza C. Chagas, Fabiano Oliveira, Alain Debrabant, Jesus G. Valenzuela, José M. C. Ribeiro, Eric Calvo

**Affiliations:** 1 Laboratory of Malaria and Vector Research, National Institute of Allergy and Infectious Diseases, National Institutes of Health, Rockville, Maryland, United States of America; 2 Laboratory of Emerging Pathogens, Center for Biologics Evaluation and Research, Food and Drug Administration, Bethesda, Maryland, United States of America; Imperial College London, United Kingdom

## Abstract

Neutrophils are the host's first line of defense against infections, and their extracellular traps (NET) were recently shown to kill *Leishmania* parasites. Here we report a NET-destroying molecule (Lundep) from the salivary glands of *Lutzomyia longipalpis*. Previous analysis of the sialotranscriptome of *Lu. longipalpis* showed the potential presence of an endonuclease. Indeed, not only was the cloned cDNA (Lundep) shown to encode a highly active ss- and dsDNAse, but also the same activity was demonstrated to be secreted by salivary glands of female *Lu. longipalpis*. Lundep hydrolyzes both ss- and dsDNA with little sequence specificity with a calculated DNase activity of 300000 Kunitz units per mg of protein. Disruption of PMA (phorbol 12 myristate 13 acetate)- or parasite-induced NETs by treatment with recombinant Lundep or salivary gland homogenates increases parasite survival in neutrophils. Furthermore, co-injection of recombinant Lundep with metacyclic promastigotes significantly exacerbates *Leishmania* infection in mice when compared with PBS alone or inactive (mutagenized) Lundep. We hypothesize that Lundep helps the parasite to establish an infection by allowing it to escape from the leishmanicidal activity of NETs early after inoculation. Lundep may also assist blood meal intake by lowering the local viscosity caused by the release of host DNA and as an anticoagulant by inhibiting the intrinsic pathway of coagulation.

## Author Summary

Salivary components from disease vectors help the arthropod to acquire blood. Here we show that an arthropod vector salivary enzyme affects the innate immune system of the host—mainly the destruction of neutrophil traps—allowing the *Leishmania* parasite to evade the host immune response and to cause an infection. This work highlights the relevance of vector salivary components in parasite transmission and further suggests the inclusion of these proteins as components for an anti-*Leishmania* vaccine. Importantly, because salivary proteins are always present at the site of natural transmission, this work further encourages the testing of vaccine candidates using the natural route of transmission—the bites of an arthropod vector—instead of current practices based solely on needle injection of parasites.

## Introduction

Leishmaniasis comprises human and animal diseases caused by parasites of the genus *Leishmania* that are transmitted by the bite of infected sand flies [1]. *Leishmania* transmission occurs when an infected sand fly probes the host's skin in search of a blood meal. During probing and feeding, sand flies salivate into the host's skin. Saliva contains powerful pharmacologic components that mediate blood-feeding success and facilitate *Leishmania* infection, first shown when *Lutzomyia longipalpis* salivary glands (SGs) were reported to enhance *Leishmania major* infection in mice [2], [3]. In the last two decades, SG and recombinant salivary proteins were investigated for their effect in enhancing pathogen transmission in different model systems (reviewed in [4]). The powerful *Lu. longipalpis* vasodilator maxadilan along with hyaluronidase were shown to facilitate transmission and establishment of *L. major* parasites [5], [6]; however, as we show here, these salivary compounds are not the only active components of sand fly saliva that exacerbate parasite infection.

Neutrophils are considered the host's first line of defense against infections and have been implicated in the immunopathogenesis of leishmaniasis [7]–[10]. *Leishmania* parasites evade killing by neutrophils by blocking oxidative burst and entering a nonlytic compartment unable to fuse with lysosomes or by resisting the microbicidal activity of neutrophil extracellular traps (NETs) [11]. The mechanism of NET formation (NETosis) in response to *Leishmania sp.* is still under investigation [11], [12]; however, recent studies have shown the direct effect of *Lu. longipalpis* SG extract (SGE) in *L. major* parasite survival inside host neutrophils [13]. This effect was abrogated by pretreatment of SGE with proteases as well as preincubation with antisaliva antibodies, supporting the hypothesis that *Lu. longipalpis* salivary protein(s) help *Leishmania* survival inside neutrophils. The negative effect of NETosis to *Leishmania* was recently documented [12].

In this report, we present direct experimental evidence that Lundep (*Lutzomyia* NET destroying protein), a secreted salivary endonuclease, is responsible for the NET-destroying activity of *Lu. longipalpis* and that this activity enhances parasite infectivity both *in vitro* and *in vivo*. Furthermore, Lundep may assist blood-meal intake by lowering the local viscosity caused by the release of host DNA and as an anticoagulant and anti-inflammatory by inhibiting the intrinsic pathway of coagulation.

## Results

### Initial findings

**Figure 1:**
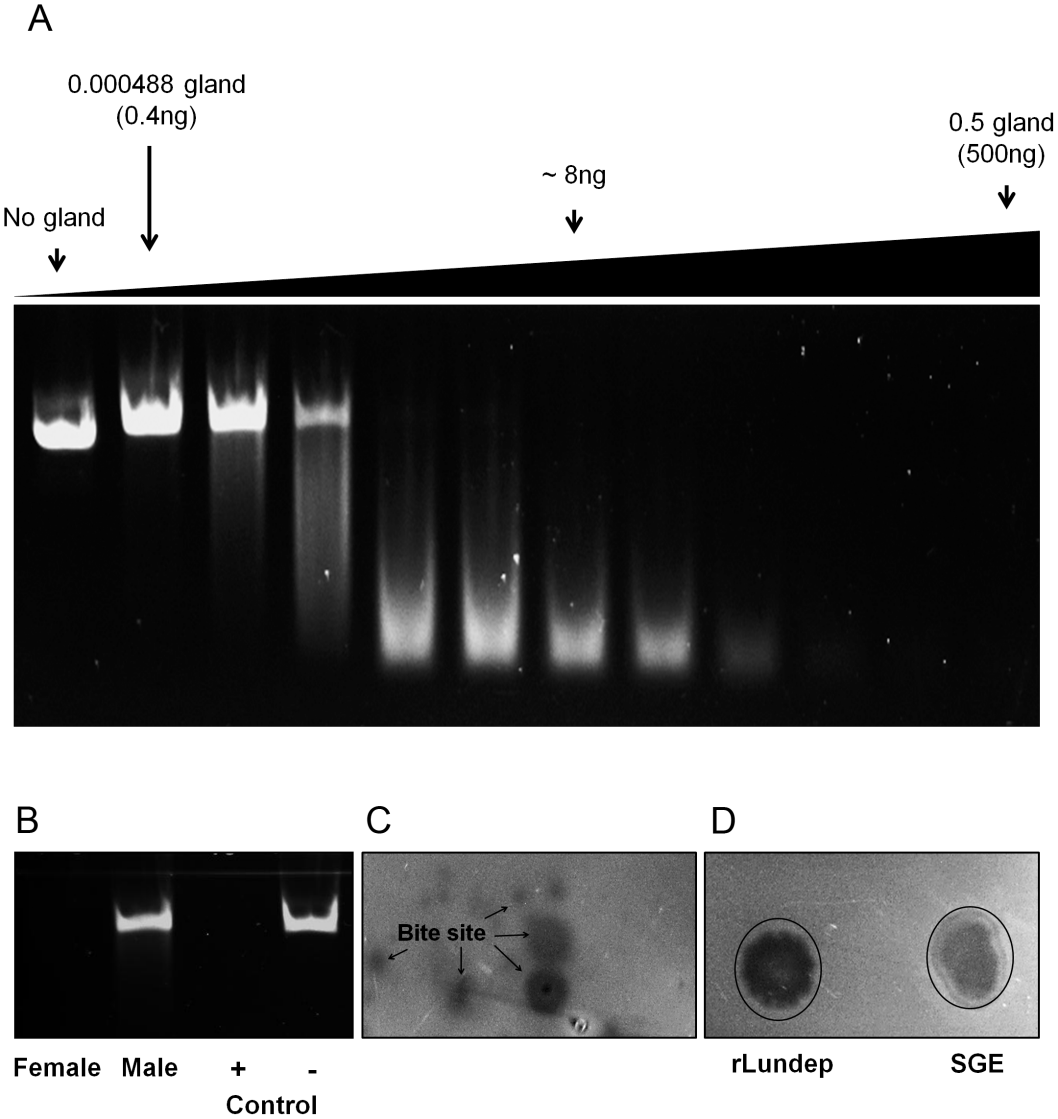
DNase activity in *Lutzomyia longipalpis* salivary gland extract (SGE) and recombinant Lundep (rLundep). (**A**) Plasmid DNA (200 µg) was incubated in a 15 µl final volume with different female SGE dilutions. After 10 minutes at 37°C, samples were electrophoresed in a 1.2% precast agarose gel and visualized under ultraviolet (UV) light. (**B**) Salivary endonuclease activity is female specific. Female and male SGE were assayed as described above. rLundep or plasmid DNA alone were used as positive and negative control, respectively. (**C**) Endonuclease is present in the saliva of female *Lu. longipalpis*. Starved sandflies were allowed to probe in an agarose gel containing plasmid DNA (200 ng/ml) supplemented with 10 mM of NHCO_3_. After 30 minutes of incubation at 37°C, the gel was stained with ethidium bromide and visualized under UV light. (**D**) One pair of SG and 1 nM of rLundep were used as a positive control.

Bioinformatic survey of the *Lu. longipalpis* sialotranscriptome [14] identified a transcript (AY455916; Lundep) containing the NUC-motif (prokaryotic and eukaryotic double (ds) and single (ss) stranded DNA and RNA endonucleases also present in phosphodiesterases) indicative of nonspecific DNA/RNA endonuclease. Alignment of the Lundep putative active center with other proteins of the same family shows the presence of the conserved RGH triad found in most DNases characterized so far (Figure S1A). The importance of these residues for catalysis has been previously studied in detail [15], [16]. Putative endonucleases retrieved using Lundep as query in the NCBI database, grouped into well supported clade, indicating that they are orthologs (Figure S1B). Visual inspection of sand fly sequences revealed the presence of signal peptide and the putative active site triade RGH necessary for DNA hydrolysis. These putative secreted salivary endonucleases may have the same biological role as Lundep in other sand fly species. The expressed sequence tag of Lundep has a predicted signal peptide of 24 aa, indicative of secretion. Accordingly, endonuclease activity was confirmed in SGEs of female *Lu. longipalpis* (Figure 1A). No endonuclease activity was detected in the SGs of males, which are not blood feeders (Figure 1B). Moreover, this activity is present in secretions of probing *Lu. longipalpis* (Figure 1C). Rabbit polyclonal antibodies against rLundep blocked the DNase activity of rLundep and SGE, indicating that Lundep is the major endonuclease in *Lu. longipalpis* SGs (Figure S2).

### Recombinant expression and purification of Lundep

Recombinant Lundep (rLundep) was cloned from a SG cDNA library using standard PCR procedures and subcloned into VR2001 expression vector. rLundep was expressed in HEK293 cells and purified by affinity and size exclusion chromatography (Figure S3A). rLundep has a strict requirement of divalent metal ions for endonuclease activity (Figure S3B,C) with broad pH optimum (5.0–8.0). Purified rLundep hydrolyzes both single-stranded (ss)- and double-stranded (ds)DNA with little sequence specificity (Figure S4). No significant RNase activity was detected (Figure S5). Lundep has a specific activity of 300,000 Kunitz U/mg as determined by a hyperchromicity assay on salmon sperm genomic DNA, where one Kunitz unit causes 0.001 change of absorbance at 260 nm per minute.

### Effect of salivary endonuclease on NET destruction and parasite survival

Because the scaffold of NETs is DNA, we hypothesized that SGE or rLundep could help *L. major* parasites escape from the microbicidal activity of NETs. To test this, we analyzed the ability of *Lu. longipalpis* SGE and rLundep to destroy human NETs induced by phorbol 12-myristate 13-acetate (PMA) and *L. major* metacyclic promastigotes.

**Figure 2:**
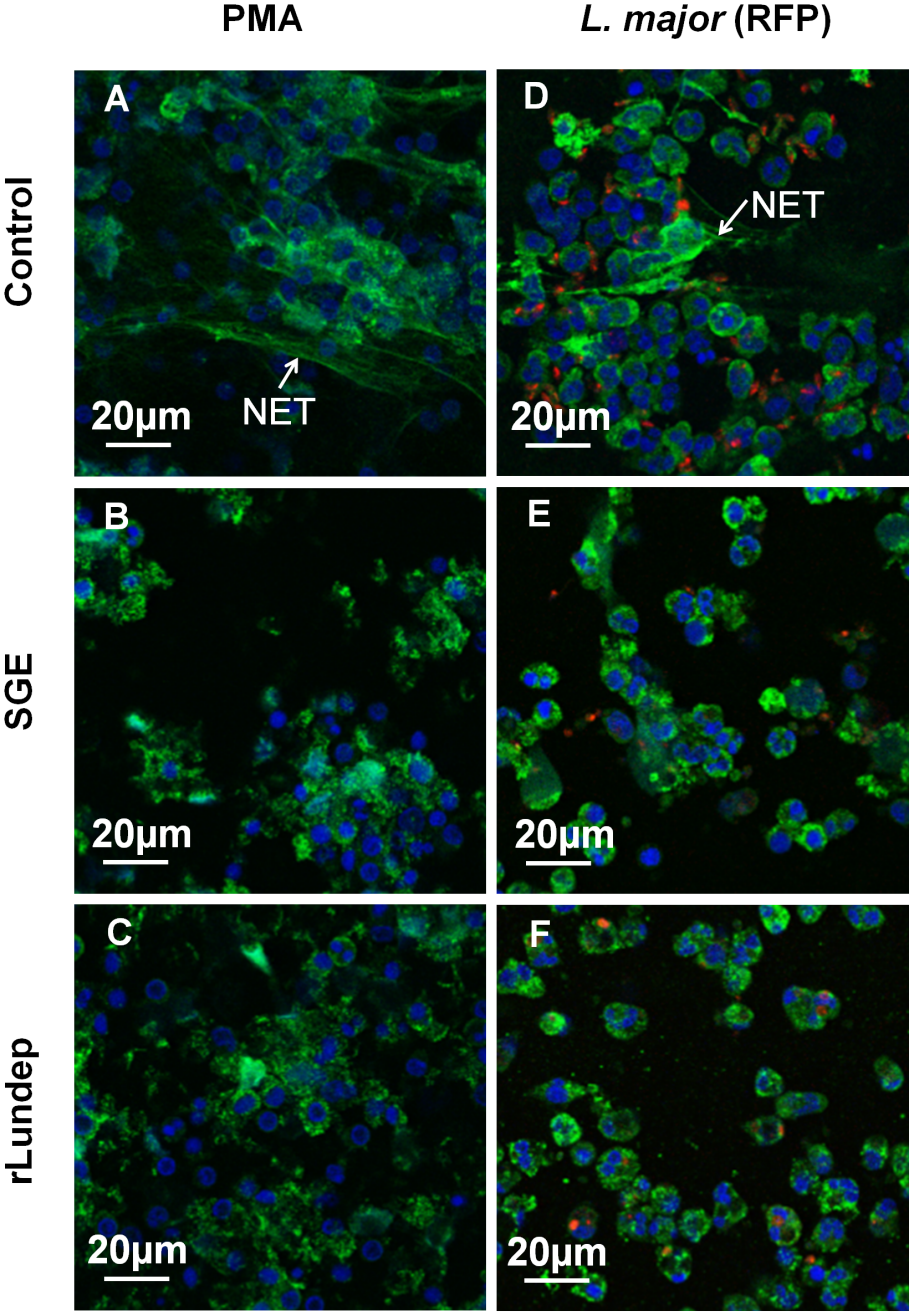
Disruption of NETs by recombinant Lundep (rLundep) and *Lutzomyia longipalpis* salivary gland extract (SGE). Human neutrophils (five healthy donors) were purified and stimulated with PMA (A–C) or 10^6^ *Leishmania major* expressing red-fluorescent protein (Lm-RFP) metacyclic promastigotes (D–F) and treated with medium (control **A**,**D**), 1 pair of *Lu. longipalpis SG* (**B**,**E**), or 3 nM of rLundep (**C**,**F**). Samples were stained for DNA (blue) and human neutrophil elastase (HNE) (green) and Lm-RFP (red). NET destruction was observed in samples treated with either *Lu. longipalpis* SG or rLundep; medium-treated neutrophils did not affect the structural integrity of NETs.

**Figure 3:**
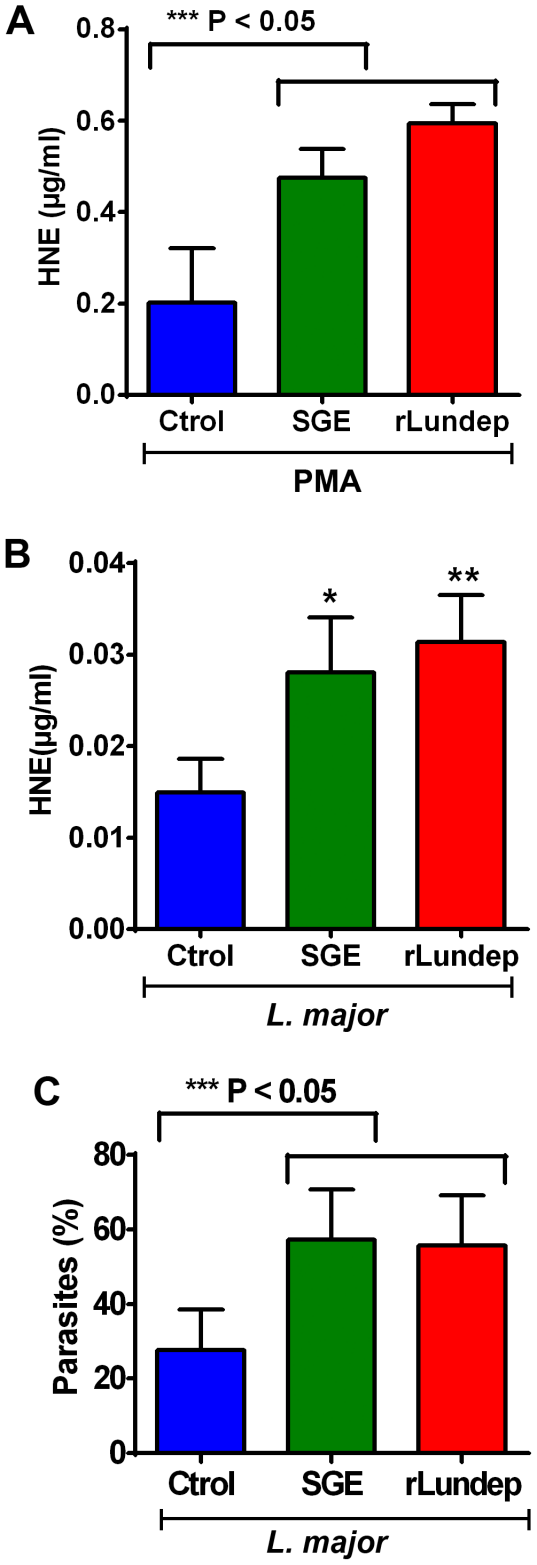
Quantification of released by activated neutrophils. (**A,B**) HNE was measured in treated supernatants using a standard curve. Measured concentrations of HNE were significantly higher in the supernatants of neutrophils treated with *Lu. longipalpis* SGE and rLundep. (**C**) Intracellular load of Lm-RFP in human neutrophils. Viable Lm-RFP promastigote counts were performed 3 days post treatment. Neutrophil infection was significantly increased in the presence of all stimuli when compared to medium (control) treated samples. The assays were performed in triplicate. Mean and SEM are shown. P<0.05 was considered statistically significant.

**Figure 4:**
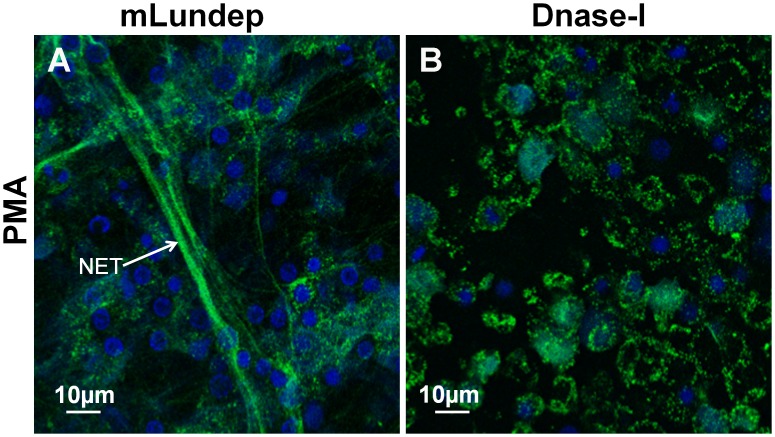
Mutagenized Lundep (mLundep) does not affect the integrity of NETs. Disruption of NETs by mLundep or commercial bovine DNase-I was investigated in PMA-induced NETs. Human neutrophils from five healthy donors were purified and stimulated with 100 nM of PMA for 4 h at 37°C. (**A**) Treatment of activated neutrophils with 200 nM of mLundep did not affect the structural integrity of NETs. (**B**) 1 U of bovine DNase-I completely hydrolyzed NETs. Cells were stained for DNA (blue) and human neutrophil elastase (HNE) (green).

Human neutrophils from healthy subjects were activated by PMA or *L. major* parasites for 4 h at 37°C. DNA—the major structural component of NETs—and neutrophil elastase (HNE) were detected by immunofluorescence (Figure 2A–F). Released human neutrophil elastase (HNE) was quantitated using a fluorogenic substrate (Figure 3A). First, we analyzed the ability of *Lu. longipalpis* SGE and rLundep to affect the integrity of PMA-induced NETs. PMA-activated neutrophils were incubated with culture medium (negative control), rLundep, *Lu. longipalpis* SG, and commercial bovine DNase-I (positive control; Figure 4). After 30 minutes of incubation, supernatants were collected for HNE quantification and neutrophils fixed and stained for DNA (blue) and HNE (green). NETs remained intact in cells treated with culture medium but were disintegrated by SG or rLundep (Figure 2B, C). Mutagenesis of Lundep active site (mLundep, RGH197AAA) abrogated its DNase activity and did not affect NETs' integrity (Figure 4). We also looked at the HNE released from NETs as an indicator of NET destruction. HNE is normally bound to NETs and found at low concentrations in culture supernatants [17]. PMA-activated neutrophils showed that treatment with SGE or rLundep significantly increases the concentration of HNE compared with control samples (Figure 3A).

The effect of rLundep and *Lu. longipalpis* SGE on *Leishmania*-neutrophil interaction was analyzed *in vitro*. Neutrophils were activated with 10^6^ *Leishmania major* expressing red-fluorescent protein (Lm-RFP) promastigotes for 4 h before treatment with *Lu. longipalpis* SG or rLundep. The NET-destroying activity of *Lu. longipalpis* SGE and rLundep hydrolyzed the parasite-induced NETs (Figure 2E,F) and significantly increased the concentration of HNE in the supernatants when compared with medium alone (Figure 3B). Furthermore, *Lu. longipalpis* SGE and rLundep significantly increased *L. major* survival, indicating that parasites can escape from the leishmanicidal activity of NETs (Figure 3C). Our results show that PMA- or *L. major* -induced NET are disrupted by treatment with commercial bovine DNase-I, *Lu. longipalpis* SGE, or rLundep (Figure 2A–F; Figure 3). These results indicate that the effect of *Lu. longipalpis* SG and rLundep in helping *L. major* parasites escape from NETs is exclusively due the catalytic activity of the salivary endonuclease.

### Lundep exacerbates *Leishmania* metacyclic promastigotes infection in mice

The infection model we used in this work is *Lu. longipalpis-L. major*. Although *Lu. longipalpis* is not the natural vector of *L. major* this specie of sand fly is permissive to *L. major* infections in laboratory conditions. Furthermore, our laboratory has a well-established murine model of infection for this pair.

**Figure 5:**
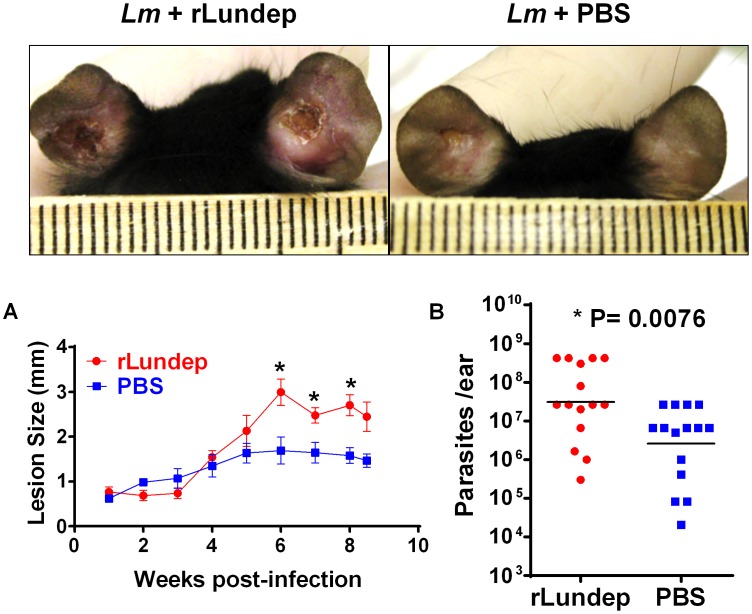
Murine cutaneous leishmaniasis model showing the *in vivo* effect of recombinant Lundep (rLundep) activity in *Leishmania major* infection. Groups of five C57BL/6 mice were inoculated intradermally in both ears with 10^3^ *L. major* in 10 µl in the presence or absence of 10 ng of rLundep. (**A**) Lesion size was measured weekly for 63 days and the mean and SEM plotted and analyzed by analysis of variance. The symbols represent the mean ± SEM. The murine cutaneous leishmaniasis model showed that co-injection of rLundep with metacyclic promastigotes significantly exacerbates *L. major* infection when compared with control. Panels reflect the pathology of the ears 9 weeks post challenge in mice coinoculated with rLundep and PBS. Ear lesions were completely healed in mice inoculated with parasite alone, while mice infected with *L. major*-rLundep had not healed after 9 weeks. (**B**) Parasite burden in lesions was evaluated by limiting dilution at the 9th week post infection. Parasite load 9 weeks after initial inoculation of *L. major* is significantly higher in mice co-inoculated with rLundep-*L. major*. Data of three independent experiments (*n* = 5) are shown.

**Figure 6:**
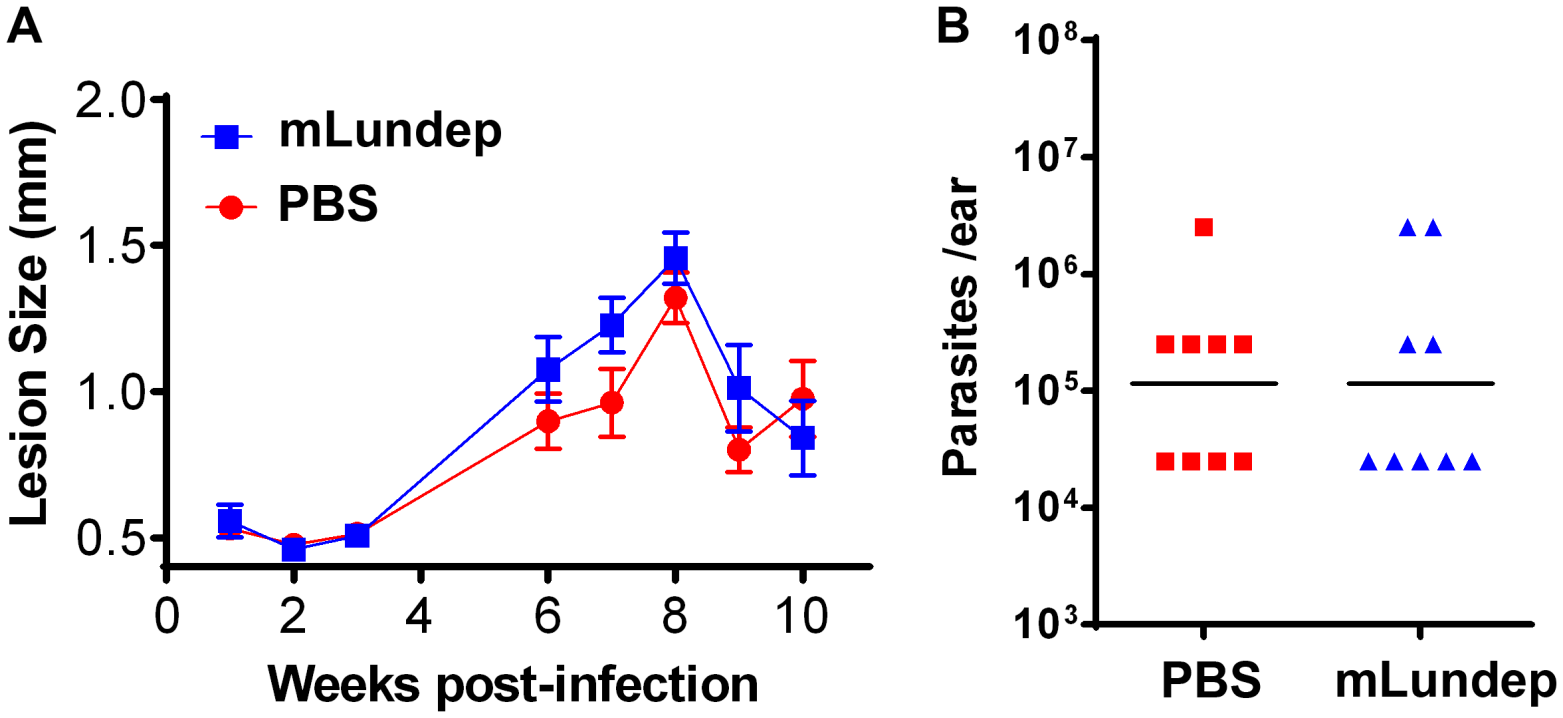
Mutation in the recombinant Lundep (rLundep) active site abrogates its virulence in a murine cutaneous leishmaniasis model. C57BL/6 mice were inoculated intradermally in both ears with 10^3^ *L. major* in 10 µl in PBS (control) or 90 ng of mutagenized Lundep (mLundep) in PBS. (**A**) Lesion size was measured for 10 weeks and the mean and SEM plotted and analyzed by analysis of variance. Symbols represent the mean ± SEM (five mice per group). The murine cutaneous leishmaniasis model showed that co-injection of mLundep with metacyclic promastigotes did not exacerbate parasite infection when compared with control. After 9 weeks, ear lesions in both groups were completely healed. (**B**) Parasite burden in lesions were evaluated by limiting dilution at the 9th week post infection. Parasite load 9 weeks after initial inoculation showed no significant differences in mice co-inoculated with mLundep when compared with control. Representative data of two independent experiments (*n* = 5) are shown.

Sand-fly bites and needle injection have been previously shown to induce neutrophil recruitment to the parasite inoculation site [18]. These neutrophils capture *L. major* parasites early after inoculation and efficiently initiate *L. major* infection. To investigate whether rLundep had any effect in exacerbating parasite infectivity *in vivo*, a model of *L. major* infection in C57BL/6 mice was utilized. Four- to five-week-old mice (five animals per group, three independent experiments) were intradermally infected with 10^3^ *L. major* promastigotes (control) or parasites admixed with rLundep. Co-inoculation of rLundep with *L. major* parasites resulted in a significantly increased cutaneous lesion, averaging 2-fold larger than those observed in the control group (Figure 5A). By week 9, control mice had their lesions significantly reduced, whereas cutaneous lesions in mice inoculated with the parasite-rLundep mixture had not healed. Furthermore, the presence of rLundep in *L. major* inoculum resulted in a markedly higher parasite burden in their lesions (15-fold) when compared with parasite alone or in the presence of mLundep (Figure 5B and Figure 6).

### Relevance of Lundep for *Lu. longipalpis* blood feeding behavior. Inhibition of the contact pathway of coagulation and feeding success

**Figure 7:**
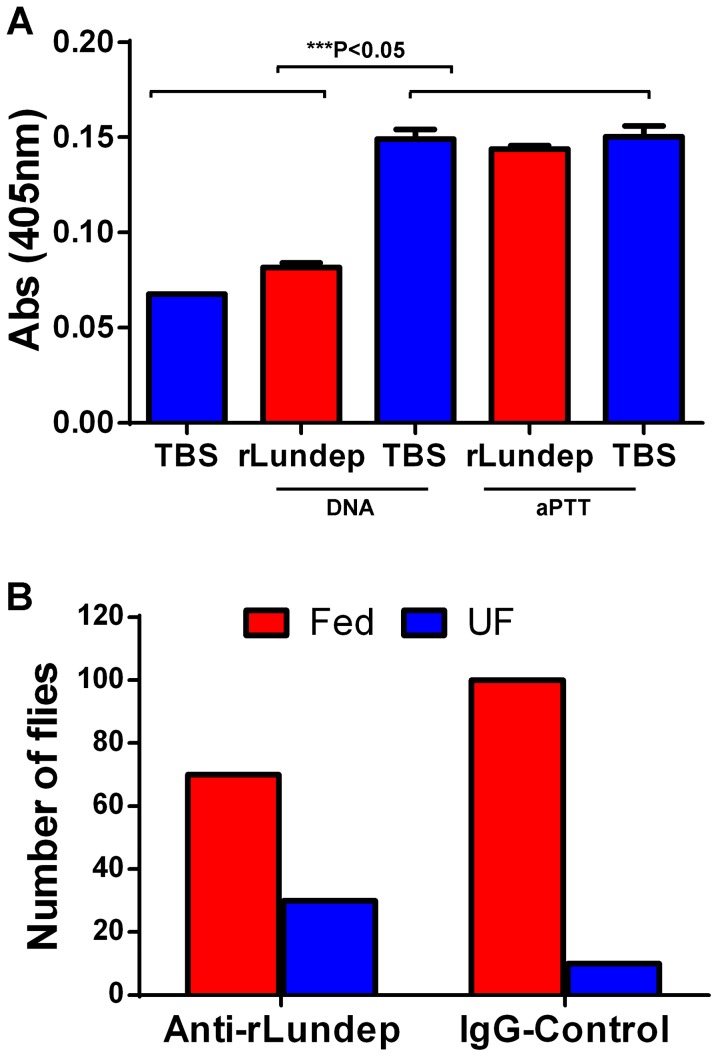
Relevance of Lundep in the blood feeding biology of *Lutzomyia longipalpis*. (**A**) Contact activation of factor XIIa by genomic DNA and aPTT reagent in human plasma was analyzed in presence or absence of Lundep. Pretreatment of DNA with Lundep abrogated its pro-coagulant activity. No effect was observed when aPPT reagent was utilized as contact activator. Enzymatic activity of factor XIIa was measure by chromogenic substrate cleavage. (**B**) Anti-Lundep antibodies significantly reduced the feeding success of sand flies in recipient mice. Sand flies were allowed to feed on passively immunized mice at 27°C in the dark. After 10 minutes, the mice were removed and the flies scored for blood meal. Four mice were used per group; the number of flies are shown as *n* in each experimental group. Results were analyzed using a χ^2^ test.

The plasma contact system consists of five plasma proteins that assemble when blood comes into contact with negatively charged surfaces. It has been previously shown that soluble DNA and NETs allow the assembly and activation of the contact system [19]. The effect of Lundep on the intrinsic coagulation pathway activation was based on the generation of human factor XIIa by soluble DNA or aPTT reagent. One hundred nM of Lundep or TBS was preincubated at 37°C with 100 µg of salmon sperm DNA or 10 µl of aPTT reagent in the presence of the chromogenic substrate S2302. After 20 minutes, the reaction was initiated by adding 50 µl of human normal reference plasma, and the amidolytic activity of FXIIa was measured at 405 nm. Figure 7A shows that pretreatment of soluble DNA with Lundep markedly inhibited activation of FXIIa in human normal reference plasma while no effect was observed when aPTT reagent was utilized. Oehmcke et al. [19] demonstrated that NETs and activated PMN cells can initiate contact activation and promote thrombus formation in the arterial and venous systems [20]. Consequently, our results indicate that the DNase activity of Lundep may contribute to the antithrombotic and anti-inflammatory functions of *Lu. longipalpis* saliva.

The feeding success of *Lu. longipalpis* on mice passively immunized with anti-Lundep or pre-immune IgG (control) was carried out on anesthetized mice. Starved female flies, 2 to 4 days old (never previously fed on blood), were placed in meshed vials, and groups of 10 flies were applied to the surface of both ears of mice passively immunized with either anti-Lundep or pre-immune (naïve) IgG. Flies were allowed to feed for 10 minutes and scored by visual inspection as fully fed, partially fed, or unfed. Figure 7B shows that flies fed on mice passively immunized with anti-Lundep antibodies were significantly less successful in obtaining a blood meal, while flies feeding on passively immunize mice with naïve IgG fed significantly better in the 10-minute period (p = 0.0001, χ^2^ test). Tripet et al. [21] highlighted the benefits of feeding aggregations in *Lu. longipalpis* in particular when feeding on hosts pre-exposed to sand flies bites, suggesting that group feeding maximizes the effect of the salivary component injected at the biting site. Accordingly, abrogating or reducing the salivary nuclease activity of the flies may result in a more viscous blood pool affecting the dispersion of other salivary components involved in blood feeding.

## Discussion

Although the killing mechanism(s) of pathogens trapped by NETs is poorly understood, the relevance of secreted endonuclease as a mechanism of evading the microorganism-killing activity of NETs has been highlighted by the presence of endonuclease activity in bacteria-evading, NET-dependent killing [17], [22]. The mechanism by which *Leishmania* promastigotes evade killing by neutrophils may be related to their ability to block oxidative burst and to enter a nonlytic compartment unable to fuse with lysosomes [23] or by resisting the microbicidal activity of NETs [11]. Munafo et al. [24] demonstrated that disrupting NETs with DNase-I attenuates extracellular production of reactive oxygen species (ROS) by neutrophils stimulated with bacteria. Moreover, this reduction in ROS production is independent of actin depolymerization and phagocytosis.

Our results demonstrate that Lundep, a female-specific secreted endonuclease, is an important factor contributing to establishment of *Leishmania* infection. Our observations are also in agreement with previous reports showing that parasite-induced NETs have leishmanicidal activity, thought to be mediated by histones, one of the NETs' structural components [12], [25]. NETs may also play a role in entrapment of parasites, hence interfering with their ability to enter host cells. Accordingly, by disrupting NETs, Lundep can effectively facilitate the survival of *L. major* parasites in neutrophils and, ultimately, in infecting macrophages and dendritic cells.

Together, these *in vitro* and *in vivo* experiments demonstrate that rLundep and *Lu. longipalpis* SG degrade the DNA scaffold of NETs, destroying their functional integrity. Furthermore, Lundep protected *L. major* parasites from the leishmanicidal activity of NETs, increasing promastigote survival and exacerbating *L. major* infection. However, we cannot exclude the possibility of an induced pathology arise from anti-NET DNAse activity or some other untested mechanism. Because Lundep exacerbated infection with *L. major in vivo* and anti-Lundep antibodies abrogate the enzyme's function, Lundep may be considered a potential vaccine target in an anti-*Leishmania* vaccine cocktail.

With regards to the role of salivary endonucleases in blood feeding arthropods, Calvo and Ribeiro [26] proposed that a salivary endonuclease from the mosquito *Culex quinquefasciatus* could act as a spreading factor for other salivary activities by reducing the local viscosity at the biting site and hence decreasing the time taken to obtain a blood meal. We also found that anti-Lundep antibodies significantly decreased the feeding success of female *Lu. longipalpis* flies in passively immunized mice. These results may have epidemiologic relevance in the potential use of Lundep in vaccine, as longer probing and feeding times may trigger defensive behavior of the host, resulting in a disruption of blood feeding or even killing of the sand fly. Evidence of cross-talking between inflammation and coagulation is mounting in the literature. Fuchs et al. [27] demonstrated that NETs are a unique link between inflammation and thrombosis-promoting thrombus organization and stability. Moreover, NETs provide a negatively charged surface that allows the binding and activation of a contact activation system. Activation of the plasma contact system triggers several cascade systems such as the kallikrein-kinin system, the intrinsic pathway of coagulation, the classical complement cascade, and the fibrinolytic system [28] rendering an unfavorable environment for blood feeding arthropods. Accordingly, hydrolyzing the DNA scaffold of NETs at the biting site may also reduce local inflammation and prevent propagation of blood clotting facilitating the intake of a blood meal.

In conclusion, we provide experimental evidence that a secreted salivary endonuclease in *Lu. longipalpis* is capable of destroying NETs produced by activated human neutrophils and that this enzymatic activity exacerbates *L. major* infection *in vivo*. Furthermore, Lundep can assist the flies in blood feeding by reducing local inflammation elicited by the vertebrate host. We believe that our findings are of broad interest to the scientific community. Besides providing new insight into the basic biology of sand-fly blood feeding, the discovery of an endonuclease in SGs of *Lu. longipalpis* may also have broad implications for understanding the biologic function of secreted endonucleases in other arthropods and the pathogens they transmit.

## Materials and Methods

Unless otherwise indicated, the protocols followed standard procedures, and all the experiments were performed at room temperature (25±1°C). All water used was of 18 MΩ quality, produced by a Milli-Q Synthesis water purification apparatus (Millipore, Billerica, MA). Hoechst 33258 dye (bis-benzamidine) was from Molecular Probes (Eugene, OR). Rabbit anti-human neutrophil elastase (HNE) was purchased from Calbiochem (San Diego, CA), and goat anti-rabbit Alexa Fluor 488 was from Invitrogen (Carlsbad, CA).

This project was approved by the Ethics Committee of the National Institutes of Health. Human neutrophils from healthy subjects were obtained under written informed consent, under NIH Clinical Center IRB-approved protocols from the NIH Clinical Center Department of Transfusion Medicine. Blood was taken from five healthy adults (aged 23–42 years).

*Lu. longipalpis* (Jacobina strain) were reared in the Medical Entomology Section, NIAID, NIH as described previously [14]. Sand flies were anesthetized with CO_2_ and transferred to a chilled plate until dissection. SGs were dissected under a stereomicroscope in 20 mM phosphate buffered saline, and SG were prepared according to Calvo and Ribeiro [29].

### Ethics statement

Public Health Service Animal Welfare Assurance #A4149-01 guidelines were followed according to the National Institute of Allergy and Infectious Diseases (NIAID), National Institutes of Health (NIH) Animal Office of Animal Care and Use (OACU). These studies were carried out according to the NIAID-NIH animal study protocol (ASP) approved by the NIH Office of Animal Care and User Committee (OACUC), with approval IDs ASP-LMVR3 and ASP-LMVR4E.

### Phylogenetic analysis of the DNA/RNA nuclease family

Blast-p analysis was performed with Lundep (AY455916) against the non-redundant database. Sequences were cleaned up to obtain a non-redundant set (proteins with >95% identity in the core domain were treated as identical) and aligned with ClustalX [30]. Alignments were manually checked, adjusted, and trimmed to include the conserved active site core. Phylogenetic analysis was performed using neighbor-joining analysis [31]. Gapped positions were treated by pairwise deletion. Poisson correction was used as a substitution model to determine pairwise distances. Confidence was determined using bootstrap analysis (10000 replicates) with 346 informative sites.

### Assay for nuclease activity

Endonuclease reactions contained 50 mM Tris, 150 mM NaCl, 5 mM MgCl_2_, pH 8.0 (TBS-M) and 200 ng double stranded (ds) circular plasmid DNA (VR2001; Vical Inc., San Diego, CA) in a final volume of 15 µl. Reaction mixtures were incubated with different dilutions of SG and recombinant Lundep. After 10 minutes at 37°C, samples were electrophoresed in 1.2% precast agarose gel (egel; Invitrogen, San Diego, CA) and visualized under ultraviolet (UV) light. RNase activity was carried out as described by Calvo and Ribeiro [29].

To demonstrate salivary secretion of endonuclease (Lundep) by *Lu. longipalpis* adult females, an *ex vivo* assay was designed. Ten female sand flies (4 days old, non-blood fed) were starved of sugar for 24 h before the test. Starved sand flies were allowed to probe for 30 minutes in a 1% agarose gel containing 50 mM Tris, 150 mM NaCl, 10 mM NaH_2_CO_3_, 1 mM MgCl_2_, pH 8.0, and 200 ng/ml ds circular plasmid DNA. The probing assay was carried out at room temperature and the probing gel kept in a slide warmer at 30°C (Precision Scientific, Chicago, IL). The combination of CO_2_ released from the bicarbonate buffer plus the temperature stimulated the sand flies to probe the slide [29]. After probing, the agarose gel was further incubated at 37°C for 30 minutes and stained with ethidium bromide. DNA hydrolysis in the gel was visualized under UV light.

### Expression and purification of Lundep

PCR fragments coding for Lundep (AY455916) were amplified (Platinum Supermix; Invitrogen) from *Lu. longipalpis* SG cDNA using gene-specific primers designed to amplify the mature peptide and added a 6x-His tag before the stop codon. PCR-amplified product was cloned into a VR2001-TOPO vector (modified version of the VR1020 vector; Vical Incorporated) and the sequence and orientation verified by DNA sequencing. Plasmid DNA (5 mg; VR2001-Lundep construct) was obtained using EndoFree plasmid MEGA prep kit (Qiagen, Valencia, CA) and filter sterilized through a 0.22-µm filter. Recombinant Lundep was produced by SAIC Advanced Research Facility (Frederick, MD) transfecting FreeStyle 293-F cells. Transfected cell cultures were harvested after 72 h and the supernatant shipped frozen to our laboratory until further processing. Recombinant protein expression was carried out by affinity and size-exclusion chromatography as described elsewhere [32]. Protein identity and purity was determined by Edman degradation and mass spectrometry.

### Western blot

rLundep (10 ng) was separated by 4–20% NuPAGE in MES buffer (Invitrogen). After electrophoresis, samples were electrotransferred onto nitrocellulose membrane using an iBlot gel transfer system (Invitrogen). The membrane was incubated overnight at 4°C with TBS (25 mM Tris, 150 mM NaCl, pH 7.4) containing 5% (w/v) powdered nonfat milk (blocking buffer), followed by incubation for 90 min at room temperature with anti-rLundep rabbit sera diluted 1∶1000 in blocking buffer. The membrane was washed 4x with TBS-T (TBS, 0.05% Tween 20) and incubated with goat anti-rabbit alkaline phosphatase conjugated (Sigma, St. Louis, MO) diluted 1∶10000 in blocking buffer. The immunoblot was developed by addition of 1 ml of Western Blue-stabilized substrate for alkaline phosphatase (Promega, Madison, WI).

### Cation dependency and pH optimum of rLundep

To determine the cation dependency of rLundep activity, reaction buffers (25 mM Tris, 150 mM NaCl) at pH 8.0 containing different divalent cations (MgCl_2_, CaCl_2_, ZnCl_2_, NiCl_2_ or CoCl_2_), 5 mM final concentration, were assayed. Reaction mixtures containing 10 nM rLundep in the appropriate buffer were incubated for 10 minutes at 37°C with 200 ng ds plasmid DNA. A negative control using 10 nM of rLundep without any ions was carried out. This negative control was also supplemented with 5 mM of EDTA to ensure no free divalent cations were present in this reaction. rLundep products were resolved in a 1.2% egel (Invitrogen) and visualized under UV light. For optimum pH, the following buffers were utilized: 50 mM sodium citrate (pH 3.0), 50 mM sodium acetate (pH 4.0 and 5.0), 50 mM MES (pH 6.0), 50 mM HEPES (pH 7 and 7.4), 50 mM Tris (pH 8.0 and 9.0), and 50 mM CAPS (pH 10.0). All reaction buffers contained 250 ng of plasmid DNA, 150 mM NaCl, and 5 mM MgCl_2_. Enzymatic reactions and DNA visualization were carried out as described above.

### Determination of rLundep substrate specificity

To determine substrate (ds or ss) specificity of rLundep, 1 nM of enzyme was incubated for 20 minutes at 37°C with different combinations of ds circular plasmid DNA or polynucleotides (ss and ds) as described [29]. Reactions were performed in TBS-M (20 µl final volume) and 400 ng of plasmid DNA or 2 µg of polynucleotides. Synthetic ds polynucleotides (1∶1 molar ratio) were produced by incubating ss-poly nucleotides for one cycle of 20 minutes at 95°C; 20 minutes at 85°C; 10 minutes at 72°C; 10 minutes at 60°C; and 10 minutes at 50°C. The pattern of nuclease activity of rLundep was determined by ion-exchange high-pressure liquid chromatography as described by Calvo and Ribeiro [29].

### DNA cleavage monitored by hyperchromicity assay

The DNA-hydrolytic activity assay was based on DNA hyperchromicity. A 1-ml quartz cuvette was loaded with 50 µg/ml DNA sodium salt from salmon testes (Sigma) dissolved in TBS-M buffer. Absorbance of the non-hydrolyzed DNA was measured before adding rLundep at three different concentrations (10, 20, and 50 nM) to the reaction cuvette. The increase of absorption of the sample at 260 nm was monitored by UV-Vis spectroscopy every 10 sec for 10 minutes. Initial velocity was calculated from slope of the linear phase of the progress curve. One Kunitz unit causes 0.001 change of absorbance at 260 nm per minute. Spectra were measured in quadruplicate in a spectrophotometer at 37°C. Identical solutions without rLundep were utilized for the blank in all cases. Spectroscopic hydrolysis analysis was performed at 37°C on a Varian Cary 100 Bio dual-beam spectrophotometer equipped with a Cary Cell Peltier temperature controller (Varian, Inc., Palo Alto, CA).

### Mutant construction, expression, and purification

Mutation for Lundep was carried out using the QuikChange I site-directed mutagenesis kit (Agilent Technologies, Santa Clara, CA) following the manufacturer's recommendations. Briefly, a high-pressure liquid chromatography-purified 40-mer complementary primer set (Lundep-RGH194AAA Forward: 5′-CTCAATTTTCTATCAGCCGCAGCTTTAAGCCCC GAAGTGG-3′ and Lundep-RGH194AAA Reverse: 3′-GGAGTTAAAAGATAGTCGGCG TCGAAATTCGGGGCTTCAC-5′) was designed to mutate R^194^, G^195^, and H^196^ in the catalytic center of Lundep for AAA (RGH194AAA, mLundep). The primers were designed to carry the triple amino-acid mutation in the central region of the oligonucleotide, flanked by 13–15 nucleotides (3′ and 5′ ends). Lundep-VR2001 plasmid (10 ng) was utilized as a template in the PCR reaction. Amplification cycles were 95°C for 1 minute followed by 18 cycles of 95°C for 50 sec, 63°C for 50 s, and 68°C for 7 minutes. After a final extension step of 10 minutes at 68°C, the PCR product was digested with 1 U of *Dnp*I to digest the parental supercoiled dsDNA. Two µl of the *Dpn*I-treated PCR product was used to transform XL10 Gold ultracompetent cells. mLundep-VR2001 plasmid DNA was isolated from transformed cells and the sequences verified by DNA sequencing. A positive plasmid construct containing the mutation was selected for expression as described above. Protein purification and DNase activity were carried out as described above.

### Isolation of human peripheral blood neutrophils

Polymorphonuclear cells were isolated from heparinized whole venous blood using Mono-Poly-Resolving Medium (MP Biomedicals, Solon, OH) according to the manufacturer's recommendations. Fresh heparinized blood was obtained from the NIH Clinical Center Department of Transfusion Medicine. The isolated fraction contained approximately 90–95% neutrophils as estimated by Trypan blue stain. The cells were counted and used immediately for NET production and visualization.

### NET production and visualization

NET production and visualization was carried out as described elsewhere [17]. Freshly purified human neutrophils (10^6^; 200-µl volume) were seeded in a Lab-Tek chamber slide (Thermo Scientific, San José, CA) for 1 h at 37°C. Seeded neutrophils were activated with 100 nM of PMA or 5×10^6^ *L. major* expressing red-fluorescent protein (Lm-RFP) metacyclic parasites for 4 h at 37°C. Activated neutrophils were individually treated with 3 nM of rLundep, 1 *Lu. longipalpis* SG pair, 1 U of bovine DNase-I (positive control), and 20 nM of mLundep or medium alone (negative control). After 1 h at 37°C, supernatants were carefully removed for HNE quantification and the cells fixed and stained for DNA and HNE as described in [17]. Images were obtained using a DMIRE2 SP2 confocal microscope (Leica, Solms, Germany). All experiments were carried out in triplicate.

### HNE measurements

HNE concentration was measured in 50 µl of supernatant using the fluorogenic substrate N-methoxysuccinyl-Ala-Ala-Pro-Val-7-amido-4-methylcoumarin (Sigma) at a final concentration of 0.25 mM (100 µl final reaction volume). The assay buffer was 50 mM HEPES buffer, pH 7.4, 100 mM NaCl, 0.01% Triton X-100. After 1 h incubation at 37°C, the substrate hydrolysis was measured in a Spectramax Gemini XPS fluorescence microplate reader (Molecular Devices, Menlo Park, CA) with 365/450 nm excitation/emission wavelengths. HNE concentration was determined by using a standard curve of serial dilutions of purified HNE (Elastin Products Company, Inc., Owensville, MO).

### Parasites

*L. major* promastigotes (WR 2885 strain) were cultured in Schneider's medium supplemented with 10% heat-inactivated fetal bovine serum, 2 mM l-glutamine, 100 U/ml penicillin, and 100 µl/ml streptomycin. WR 2885 strain was typed at the Walter Reed Army Institute of Research [33]. Infective-stage metacyclic promastigotes of *L. major* were isolated from stationary cultures (4–5 days old) by negative selection using peanut agglutinin (Vector Laboratories, Inc., Burlingame, CT). Metacyclic promastigotes (1000) with or without 10 ng of rLundep in 10 µl of PBS buffer (supplemented with 5 mM of MgCl_2_) were inoculated intradermally into both ears' dermis using a 29-gauge needle. Evolution of the lesion was monitored weekly by measuring ear thickness using a vernier caliper (Mitutoya America Corporation, Aurora, IL).

### Measurement of parasite loads in human neutrophils

Human neutrophil from five healthy donors (2×10^6^) were infected with 10^7^ Lm-RFP metacyclic parasites. After 4 h of incubation at 37°C, samples were treated with rLundep (100 nM), 1 SG pair of *Lu. longipalpis*, 1 U of bovine DNase-I (positive control) or medium alone (negative control). After 3 days of incubation at 23°C, cells were harvested and the supernatants spun down to evaluate the viable parasites. Live parasites were stained with Giemsa. All experiments were carried out in triplicates.

### Generation of Lm-RFP parasites

A region containing the DsRed gene flanked by the 5′ and 3′ untranslated regions of the *Leishmania donovani* A2 gene was amplified by PCR using the following forward A2F and reverse A2R primers and the pKSNEO-DsRed plasmid as template [34]. The A2F primer 5′-TGGCATATGCGTCGACCGCTGCTTGCGTTC-3′ contains a *Sal*I restriction site. The A2R primer 5′-ACGCGTGGATCCTGAATTCGAGCTCTGGAGAGA-3′ contains a *BamH*I restriction site. The resulting ∼3.7-kb PCR fragment was cloned and sequenced. It contained an internal *BamH*I site that was mutated using standard PCR techniques. The approximately 3.7-kb *Sal*I/*BamH*I fragment with the mutated internal *BamH*I site was subsequently cloned into the *Sal*I and *BamH*I sites of the pF4X1.4hyg plasmid (Jena Biosciences, GmbH, Jena, Germany), resulting in the pA2RFPhyg plasmid. This plasmid was digested with *Swa*I to generate a linear fragment containing the RFP/Hyg expression cassette flanked by the 5′ and 3′ ssu sequences used for homologous recombination into the parasite 18S rRNA gene locus (ssu locus) as described in the original pFX1.4hyg plasmid (Jena Biosciences).

Promastigotes of the *L. major* Friedlin strain, NIH/FV1 (MHOM/IL/80/FN) were transfected by electroporation with 20 µg of *Swa*I-digested pA2RFPhyg plasmid as described previously [35] and plated onto 1% noble agar plates prepared in M199 *Leishmania* culture medium [36] supplemented with 4 µg/ml 6-biopterin (Calbiochem) and 100 µg/ml hygromycin B (Roche). Integration of the RFP/Hyg cassette into the ssu locus of hygromycin B-resistant clones was confirmed by PCR.

### Measurement of parasite loads

Parasite load was determined using a limiting dilution assay as described elsewhere [37]. Briefly, ear tissue was excised and homogenized in RPMI medium. The homogenate was serially diluted on Schneider medium 10% heat-inactivated fetal bovine serum, 2 mM l-glutamine, 100 U/ml penicillin, 100 µl/ml streptomycin, and 40 mM HEPES and seeded in 96-well plates containing biphasic blood agar (Novy-Nicolle-McNeal). The number of viable parasites was determined from the highest dilution at which promastigotes could be found after 21 days of culture at 23°C.

### Polyclonal antibody production and purification

Polyclonal antibodies against rLundep were raised in rabbits by Spring Valley Laboratories, Inc. (Woodline, MD) using a standard protocol. Briefly, rabbits were immunized three times with 125 µg of rLundep every 21 days and the serum collected at day 72. A 10-ml aliquot of rabbit serum (immunized or naïve) was diluted to 50 ml in phosphate buffer, pH 6.5, and loaded onto a 5-ml HiTrap protein A HP column (GE Healthcare, Piscataway, NJ) and the IgG eluted using a linear gradient of citric acid (100 mM, pH 3.4) on an Akta purifier system (GE Healthcare). Fractions containing purified IgG were pooled and dialyzed against 1× PBS for 16 h at 4°C. IgG quantification was based on 1 absorbance unit at 280 nm equals 0.7 mg/ml.

### Antibody protection assay

To investigate the neutralizing activity of anti-rLundep antibodies on its enzymatic activity, *an in vitro* assay was developed. Purified anti-rLundep or naïve antibodies (0 and 5 µg/ml) were mixed with rLundep (10 nM) or SGEs (1 pair) and incubated for 30 minutes at 37°C. Plasmid DNA hydrolysis and visualization was carried out as described above.

### Effect of rLundep on the intrinsic pathway of blood coagulation

The effect of rLundep on the intrinsic coagulation pathway was based on the generation of human factor XIIa by DNA or aPTT reagent. Ten µl of rLundep (100 nM) or TBS was preincubated at 37°C with 100 µg of salmon sperm DNA (Sigma) or 10 µl of aPTT reagent (Helena Laboratories, Beaumont, TX) in the presence of 5 µl of the chromogenic substrate S2302 (Diapharma, West Chester, OH). TBS alone was utilized as the reaction blank. After 20 minutes, the reaction was initiated by adding 50 µl of citrated-human normal reference plasma (Diagnostica Stago, Inc. Parsippany, NJ) and the amidolytic activity of fXIIa measured at 405 nm in a plate reader (Molecular Devices). All reactions were supplemented with 5 mM MgCl_2_. Final concentrations of rLundep and S2302 in the assay reaction were 13 nM and 300 µM, respectively.

### Feeding success in passively immunized mice

Purified rabbit anti-Lundep or naïve antibodies were given to the recipient mice via intraperitoneal inoculation of 100 µg (100 µl volume) 15 minutes before exposing five to six CL57B/6 mice to sand flies (10 flies in each ear). Feeding success of sand flies in passively immunized mice was measured on anesthetized animals (four per group) as described Belkaid et al. [38]. Briefly, groups of 10 female flies (3 to 4 day old) were caged in polystyrene tube the day before the experiments was carried out and deprived of sugar. Ready-to-use vials containing starved flies were applied to the surface of anesthetized mouse ear that was previously given either anti-Lundep antibodies or purified naïve IgG. Flies were allowed to feed for 10 min, removed, and scored as either fed or unfed. Flies with their entire abdomen fully engorged or with visible blood were considered as fed. *Lu. longipalpis* flies used in this experiment were not blood fed previously and had no eggs in their abdomen, enabling assessment of a blood meal by visual inspection.

### Statistical analysis

Data were analyzed using GraphPad Prism v 5 software (GraphPad Software, Inc., San Diego, CA) and plotted as bar graphs or scatter plots. Comparisons were made with the 2-tailed *t* test with 95% confidence interval and 2-way analysis of variance. P<0.05 was considered significant (*, p<0.05; **, p<0.01; ***, p<0.001).). For feeding success, a χ^2^ test with 95% confidence interval was used.

## Supporting Information

Figure S1 **Bioinformatic analysis of Lundep.** (**A**) Amino acid (aa) sequence alignment of the putative active site conserved elements among different endonucleases. Most biochemically characterized endonucleases have the conserved R(K)GH triad. Lundep also contains other aa residues (highlighted in red) implicated in the nucleophilic attack of DNA substrate and stabilization of the active site. (**B**) Phylogenetic analysis of endonucleases. Protein sequences were aligned by the ClustalW program (DNAstar). The unrooted neighbor-joining tree (10,000 bootstraps) was generated by MEGA 5.05 software. The numbers on the tree bifurcations indicate the percentage bootstrap support above 75%. The bar at the bottom represents 10% aa substitution. CuQuEnd: *Culex quinquesfaciatus*, Smarcens: *Serratia* marcescens, Pcamtsch: *Paralithodes camtschaticus*, Hsapiens: *Homo sapiens*.
(TIF)

Figure S2 **Polyclonal antibodies against recombinant Lundep (rLundep) abrogate the endonuclease activity of rLundep and salivary gland extract (SGE).** The ability of rabbit anti-rLundep antibodies to neutralize the enzymatic activity of Lundep was studied using an *in vitro* assay. rLundep (10 nM) or 1 pair of *Lutzomyia longipalpis* SG were preincubated at 37°C with 1 µg of protein A-purified rabbit anti-rLundep or naïve antibodies. After 30 minutes, 200 ng of plasmid DNA in TBS-M was added to each reaction and further incubated for 10 minutes at 37°C. Reactions were electrophoresed in a 1.2% precast agarose gel and visualized under ultraviolet light.
(TIF)

Figure S3 **Biochemical characterization of recombinant Lundep (rLundep).** (**A**) Purification of rLundep after HiTrap chelating column and size-exclusion chromatography. Inset: NuPAGE of purified rLundep (**a**) under reducing conditions and western blot detection (**b**) of rLundep using rabbit polyclonal anti-rLundep antibodies. Molecular standard was SeeBlue Plus2 in kDa. Control without ions (-ions) also contained 5 mM EDTA. (**B**) DNA hydrolysis by rLundep is magnesium dependent. (**C**) Effect of pH on the DNase activity of rLundep exhibited a broad pH optimum (5.0–8.0).
(TIF)

Figure S4 **Substrate specificity of recombinant Lundep (rLundep).** Separation of rLundep hydrolysis products and standards was carried out on a TSKgel DEAE column. Substrate specificity of rLundep was determined using plasmid DNA or different synthetic ds- and ss-DNA substrates. Recombinant Lundep hydrolyzed ss- and dsDNA with some sequence specificity. (**A**) Poly T standard used to calibrate the column. Numbers in peaks represent ss-polymer nucleotide size. (**B**) rLundep hydrolyzes plasmid DNA, indicating its endonuclease activity. (**C**) rLundep hydrolyzes both ss- and dsDNAs (50mer) almost indiscriminately. (**D**) No hydrolysis was found toward 10mer hetero or homo purine-purine and pyrimidine-pyrimidine polymers. (**E**) rLundep hydrolyzed 10mer purine-pyrimidine duplex. (**F**) rLundep alone. Hydrolysis products, when they occurred, ranged in size from 4 to 10 nucleotides. Representative chromatograms are shown.
(TIF)

Figure S5 **Recombinant Lundep (rLundep) and** ***Lutzomyia longipalpis*** **salivary glands (SGs) show a marginal RNase activity.** Yeast RNA was incubated with rLundep or *Lu. longipalpis* SGs for 20 minutes in TBS-M at 37°C. Samples were electrophoresed in a 2% precast agarose gel and visualized under ultraviolet light. One unit of commercial RNase-A was used as a positive control.
(TIF)
