# Supplementary figures and images for: Lundep, a Sand Fly Salivary Endonuclease Increases Leishmania Parasite Survival in Neutrophils and Inhibits XIIa Contact Activation in Human Plasma
Source: PLoS Pathog. 2014 Feb 6;10(2):e1003923. doi: 10.1371/journal.ppat.1003923 (PMC3916414; doi:10.1371/journal.ppat.1003923)

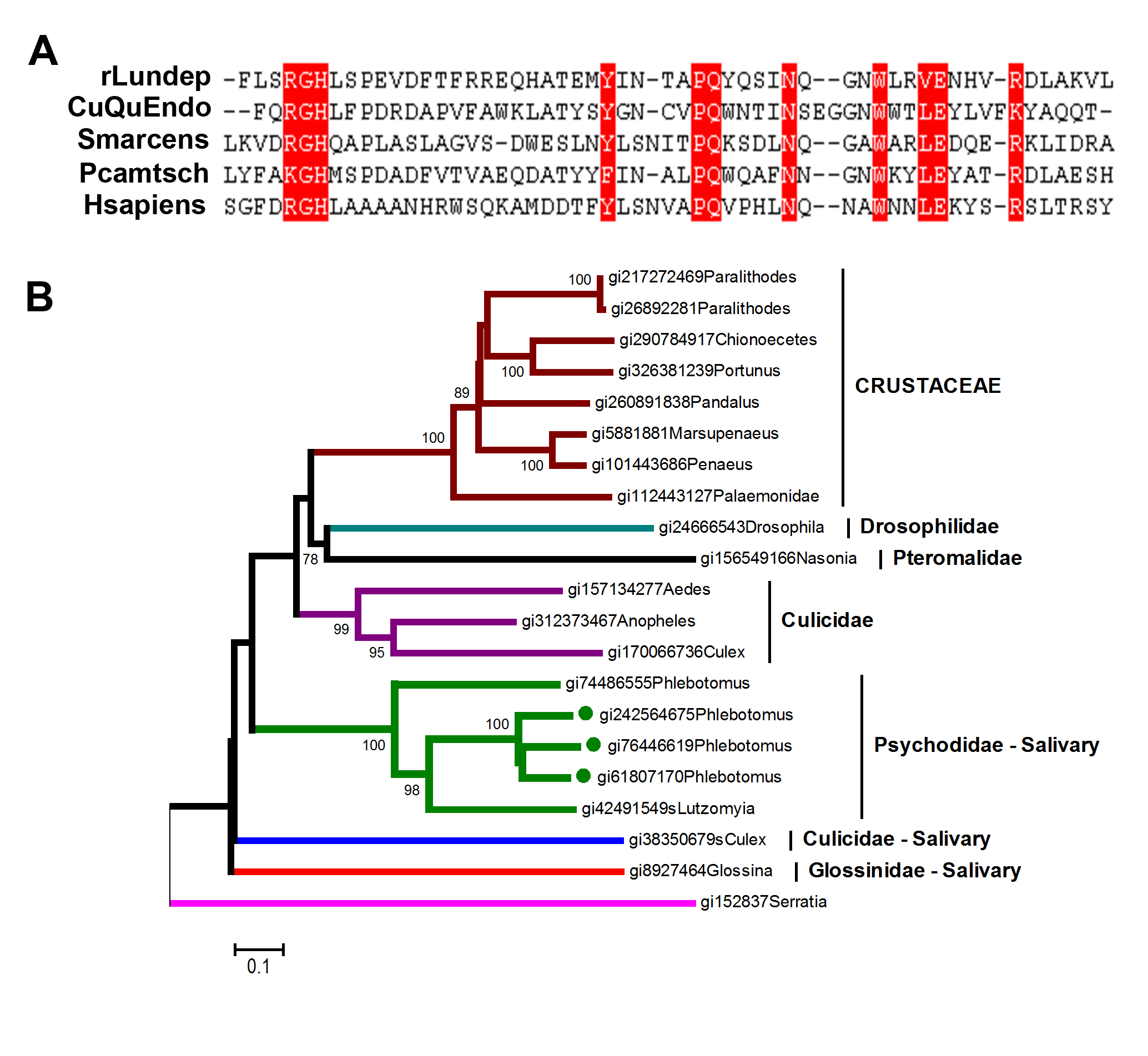

Supplement: Figure S1 — Bioinformatic analysis of Lundep. (A) Amino acid (aa) sequence alignment of the putative active site conserved elements among different endonucleases. Most biochemically characterized endonucleases have the conserved R(K)GH triad. Lundep also contains other aa residues (highlighted in red) implicated in the nucleophilic attack of DNA substrate and stabilization of the active site. (B) Phylogenetic analysis of endonucleases. Protein sequences were aligned by the ClustalW program (DNAstar). The unrooted neighbor-joining tree (10,000 bootstraps) was generated by MEGA 5.05 software. The numbers on the tree bifurcations indicate the percentage bootstrap support above 75%. The bar at the bottom represents 10% aa substitution. CuQuEnd: Culex quinquesfaciatus, Smarcens: Serratia marcescens, Pcamtsch: Paralithodes camtschaticus, Hsapiens: Homo sapiens. (TIF) [file ppat.1003923.s001.tif]

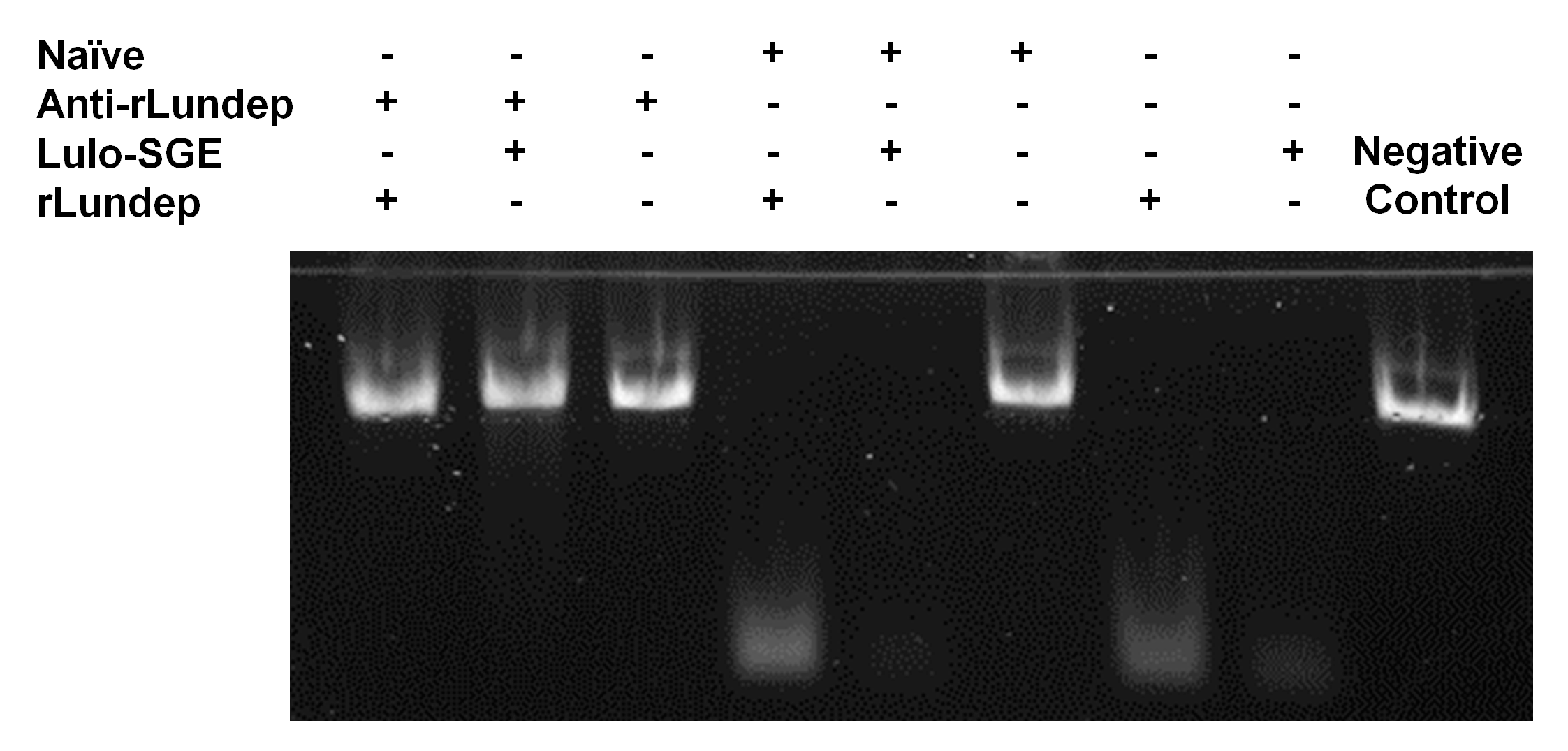

Supplement: Figure S2 — Polyclonal antibodies against recombinant Lundep (rLundep) abrogate the endonuclease activity of rLundep and salivary gland extract (SGE). The ability of rabbit anti-rLundep antibodies to neutralize the enzymatic activity of Lundep was studied using an in vitro assay. rLundep (10 nM) or 1 pair of Lutzomyia longipalpis SG were preincubated at 37°C with 1 µg of protein A-purified rabbit anti-rLundep or naïve antibodies. After 30 minutes, 200 ng of plasmid DNA in TBS-M was added to each reaction and further incubated for 10 minutes at 37°C. Reactions were electrophoresed in a 1.2% precast agarose gel and visualized under ultraviolet light. (TIF) [file ppat.1003923.s002.tif]

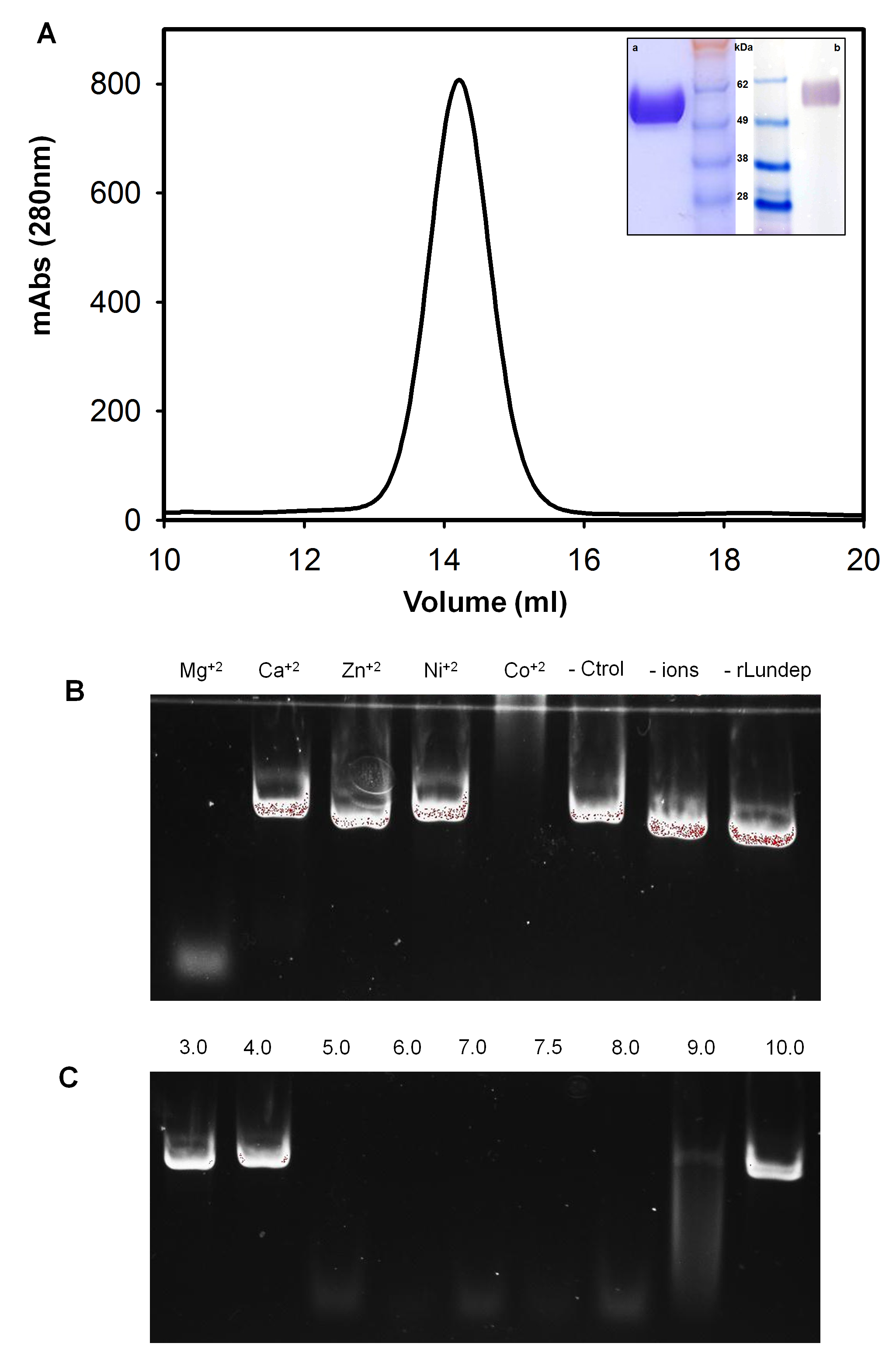

Supplement: Figure S3 — Biochemical characterization of recombinant Lundep (rLundep). (A) Purification of rLundep after HiTrap chelating column and size-exclusion chromatography. Inset: NuPAGE of purified rLundep (a) under reducing conditions and western blot detection (b) of rLundep using rabbit polyclonal anti-rLundep antibodies. Molecular standard was SeeBlue Plus2 in kDa. Control without ions (-ions) also contained 5 mM EDTA. (B) DNA hydrolysis by rLundep is magnesium dependent. (C) Effect of pH on the DNase activity of rLundep exhibited a broad pH optimum (5.0–8.0). (TIF) [file ppat.1003923.s003.tif]

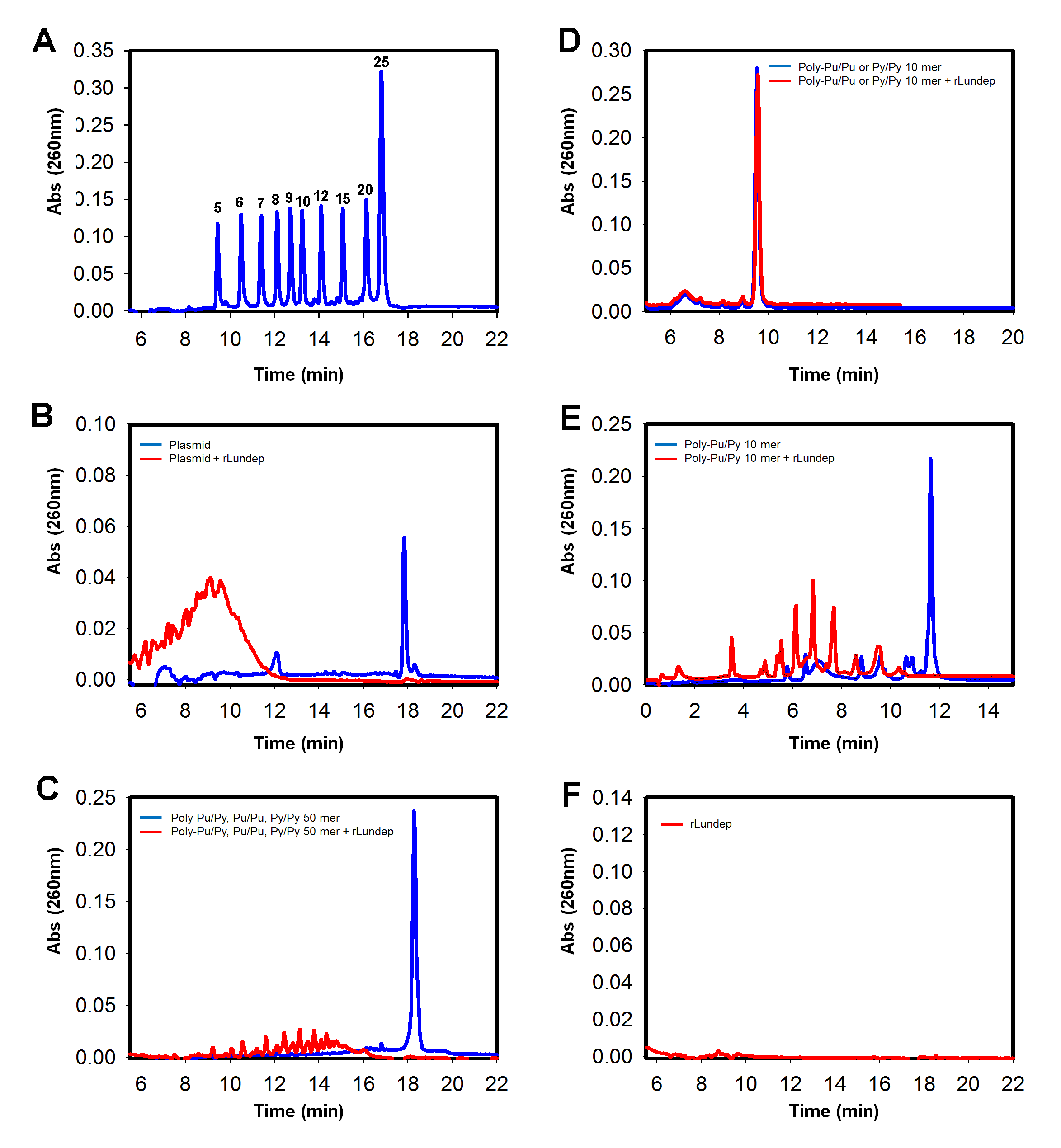

Supplement: Figure S4 — Substrate specificity of recombinant Lundep (rLundep). Separation of rLundep hydrolysis products and standards was carried out on a TSKgel DEAE column. Substrate specificity of rLundep was determined using plasmid DNA or different synthetic ds- and ss-DNA substrates. Recombinant Lundep hydrolyzed ss- and dsDNA with some sequence specificity. (A) Poly T standard used to calibrate the column. Numbers in peaks represent ss-polymer nucleotide size. (B) rLundep hydrolyzes plasmid DNA, indicating its endonuclease activity. (C) rLundep hydrolyzes both ss- and dsDNAs (50mer) almost indiscriminately. (D) No hydrolysis was found toward 10mer hetero or homo purine-purine and pyrimidine-pyrimidine polymers. (E) rLundep hydrolyzed 10mer purine-pyrimidine duplex. (F) rLundep alone. Hydrolysis products, when they occurred, ranged in size from 4 to 10 nucleotides. Representative chromatograms are shown. (TIF) [file ppat.1003923.s004.tif]

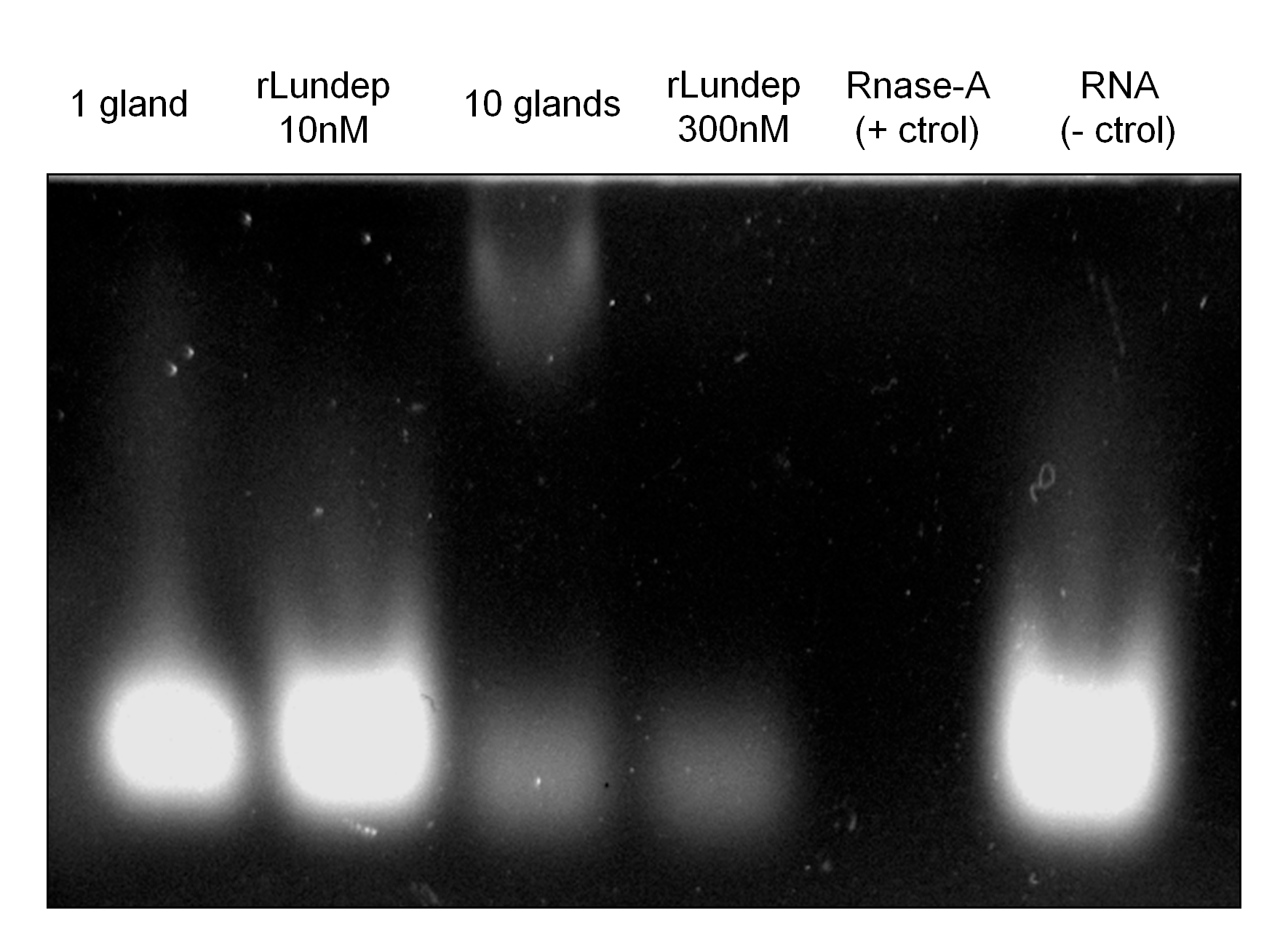

Supplement: Figure S5 — Recombinant Lundep (rLundep) and Lutzomyia longipalpis salivary glands (SGs) show a marginal RNase activity. Yeast RNA was incubated with rLundep or Lu. longipalpis SGs for 20 minutes in TBS-M at 37°C. Samples were electrophoresed in a 2% precast agarose gel and visualized under ultraviolet light. One unit of commercial RNase-A was used as a positive control. (TIF) [file ppat.1003923.s005.tif]
